# A deep learning-based dynamic deformable adaptive framework for locating the root region of the dynamic flames

**DOI:** 10.1371/journal.pone.0301839

**Published:** 2024-04-17

**Authors:** Hongkang Tao, Guhong Wang, Jiansheng Liu, Zan Yang

**Affiliations:** 1 School of Advanced Manufacturing, Nanchang University, Nanchang, China; 2 Research Center of Manufacturing Industry Information Engineering Technology of Jiangxi Province, Nanchang, China; 3 Jiangxi Tellhow Military Industry Group Co., Ltd., Nanchang, China; Bahria University - Lahore Campus, PAKISTAN

## Abstract

Traditional optical flame detectors (OFDs) in flame detection are susceptible to environmental interference, which will inevitably cause detection errors and miscalculations when confronted with a complex environment. The conventional deep learning-based models can mitigate the interference of complex environments by flame image feature extraction, which significantly improves the precision of flame recognition. However, these models focus on identifying the general profile of the static flame, but neglect to effectively locate the source of the dynamic flame. Therefore, this paper proposes a novel dynamic flame detection method named Dynamic Deformable Adaptive Framework (DDAF) for locating the flame root region dynamically. Specifically, to address limitations in flame feature extraction of existing detection models, the Deformable Convolution Network v2 (DCNv2) is introduced for more flexible adaptation to the deformations and scale variations of target objects. The Context Augmentation Module (CAM) is used to convey flame features into Dynamic Head (DH) to feature extraction from different aspects. Subsequently, the Layer-Adaptive Magnitude-based Pruning (LAMP) where the connection with the smallest LAMP score is pruned sequentially is employed to further enhance the speed of model detection. More importantly, both the coarse- and fine-grained location techniques are designed in the Inductive Modeling (IM) to accurately delineate the flame root region for effective fire control. Additionally, the Temporal Consistency-based Detection (TCD) contributes to improving the robustness of model detection by leveraging the temporal information presented in consecutive frames of a video sequence. Compared with the classical deep learning method, the experimental results on the custom flame dataset demonstrate that the AP_0.5_ value is improved by 4.4%, while parameters and FLOPs are reduced by 25.3% and 25.9%, respectively. The framework of this research extends applicability to a variety of flame detection scenarios, including industrial safety and combustion process control.

## 1 Introduction

The lives and property of individuals are directly impacted by fire safety. In complex outdoor environments, the rapid spread of fire will cause casualties and property loss. Thus, timely detection of flame becomes exceptionally crucial. Optical flame detectors (OFDs) employ sensors to monitor specific light frequencies for flame detection. OFDs are capable of successfully fusing particular feature fusion algorithms, such as scale invariant feature transform (SIFT) [1], flame detection algorithm based on multi-feature fusion (FDAMF) [2], and fire color feature extraction (FCFE) [3], with optical sensor hardware, such as [4, 5], and so on. These OFDs, i.e., infrared [6], ultraviolet [7], and infrared/ultraviolet [8], are frequently used in the community of firefighting [9], medical [10], and agricultural [11]. OFDs integrate optical sensors and algorithms for flame detection in diverse applications. Naturally, the valuable signals obtained from optical sensors in OFDs can be effectively combined by these feature fusion algorithms, enhancing the overall capability of flame detection systems.

However, the OFDs face limitations regarding their detection process, susceptibility to interference in complex environments, and compatibility with advanced devices. For the first one, due to certain characteristics such as signal transmission and communication interface requirements, OFDs adopt multi-stage detection methods. These methods include sequential processes like feature detection, feature-to-signal transformation, and target recognition, which collectively lead to a low detection speed. For the second one, false detections in outdoor environments may be influenced by objects with similar flame characteristics. (e.g., red roses, fire engine, sun, and among others). For the third one, the algorithms of traditional OFDs may struggle to effectively locate the root region of dynamic flames that exhibit rapid changes in shape, intensity, or movement. Eventually, these computationally complex algorithms of OFDs are not well-suited for contemporary advanced devices, leading to a sluggish device detection process.

To overcome the low robustness and slow detection speed in the existing OFDs, the one-stage deep learning (DL)-based object detection methods, which recognize objects directly, have gained wide attention. They are effective in many fields, such as intelligent security [12], intelligent transportation [13], and intelligent firefighting [14]. Specifically, the DL model automatically discovers and extracts the most relevant and salient features of each object class through neural networks training data [15]. Moreover, DL-based methods are generally end-to-end models, i.e. [13, 16, 17], where the target features are directly extracted from the input data, and thus the computational efficiency is greatly improved significantly. Currently, the availability of abundant computational resources, such as GPU and TPU, enables faster inference speeds for deep learning algorithms, making their application on devices feasible [18]. Therefore, Deep learning-based methods are more compatible for integration with existing optical sensing devices. Additionally, DL-based methods are becoming increasingly mature in various industrial applications [19, 20], further confirming the effectiveness of such methods.

In many studies, the You Only Look Once (YOLO) family of DL-based algorithms [21–23], such as YOLOv5 [24], possess characteristics of end-to-end and multi-scale object detection. These characteristics render it exceptional in terms of the level of speed and real-time detection. However, when faced with flame detection, DL-based YOLO has limitations in real-time detecting dynamic flames efficiently where the landscape of the dynamic flame is uncertain. Concretely, the current DL-based YOLO in flame recognition has primarily focused on balancing model accuracy and efficiency, often overlooking the enhancement of robustness in flame detection models. More importantly, recent studies [25, 26] suggest that the robustness of object detection models is a critical factor in designing new models, however, the existing flame detection methods have not explicitly considered how to improve the robustness. Thus, the research on flame detection has not been designed based on the two types of key features of flame, i.e., dynamic recognition and flame root region localization. Therefore, the deformable convolution network v2 (DCNv2) [27], context augmentation module (CAM) [28], dynamic head (DH) [29], and temporal consistency-based detection (TCD) [30], are effectively integrated into YOLOv5 to address these challenges. Specifically, DCNv2 employs deformable convolutional offsets to enhance the extraction of flame image features across different scales. CAM employs an adaptive fusion method to effectively combine the features obtained from multi-scale expanded convolutions. DH integrates three self-attention mechanisms, i.e., scale-aware, spatial-aware, and task-aware, to generate real-time results. TCD determines whether targets are real or not by focusing on their consistency across consecutive frames. These techniques mitigate the complexities of the environment effectively and enhance the precision of dynamic flame detection significantly. After integrating the four techniques, the pruning technique [31] is introduced to accelerate the detection speed. Then the ability to localize the flame root region has been improved by the inductive modeling (IM) approach [32, 33] when the precision is enhanced. Research findings indicate that targeting the flame root region for fire suppression enables more effective control of the fire [34–36]. Therefore, this paper proposes a novel dynamic flame detection method named Dynamic Deformable Adaptive Framework (DDAF) for locating the flame root region dynamically.

The main contributions of this paper can be summarized as follows.

1. The proposed Dynamic Deformable Adaptive Framework achieves the localization of the flame root region by detecting the flame. This improvement is essential for achieving more effective fire suppression and control.
2. To overcome the limitations of existing algorithms in OFDs, this paper introduces four techniques i.e., deformable convolution network v2 (DCNv2), context augmentation module (CAM), dynamic head (DH), and temporal consistency-based detection (TCD), and integrate them into YOLOv5 to improve dynamic feature extraction capability and detection robustness. These techniques enhance the precision of real-time detection for dynamic flames, even in uncertain flame landscape conditions.
3. In this study, a pruning technique is applied to remove redundant parameters from the model, thus improving the flame detection speed.
4. This paper successfully combines the inductive modeling (IM) method with YOLOv5 to model the flame landscape, enabling the effective location of the flame root region.

The remainder of this paper is organized as follows. Section 2 briefly introduces the related work and background technique. Section 3 introduces the DDAF. Section 4 introduces the experimental results and analysis. Finally, Section 5 concludes this paper.

## 2 Related works and background techniques

### 2.1 Related works

This study builds on several previous works: conventional algorithms in OFDs, the YOLO-based end-to-end algorithms, and object detection algorithms for temporal consistency. The YOLO-based end-to-end algorithms have successfully tackled the limitations of conventional algorithms in the OFDs, and the TCD technique offers a solution to address the specific limitations of the YOLO-based method in flame detection.

OFD is a significant piece of devices for ensuring fire safety. The algorithms integrated into OFDs play a crucial role in enhancing their flame detection capabilities. During the past decades, various algorithms for OFDs have been proposed, including those based on infrared [4, 6, 37], ultraviolet [7, 38, 39], and infrared/ultraviolet [5, 8, 40] technologies. Liu et al. proposed a detector of flame/smoke video image detection system consisting of one infrared camera and others [6]. Truong et al. proposed a low-cost and reliable smart fire alarm system that utilizes ultraviolet detection technology [7]. Genovese et al. proposed an image processing system for the detection of wildfire smoke based on computational intelligence techniques by infrared/ ultraviolet cameras [8]. They all detect flames by the characteristic wavelengths of the fire. These wavelengths are transformed into characteristic signals. Then, the detector combines the signals from both sensors and is used to determine if the target is a flame based on a pre-defined threshold. However, the quantification of flame features with two-dimensional data [41] and dependence on threshold settings [6] will result in limited detection speed and inadequate adaptability for different environments respectively. This highlights the need for advancements in flame detection technologies, motivating the exploration of alternative approaches.

Over the years, the YOLO [21–23, 42–44] family has been one of the popular one-stage real-time object detectors. YOLO detectors can be found in many hardware platforms and application scenarios, meeting different requirements. After years of development, YOLO has evolved into a series of high-speed models demonstrating strong performance. Compared to multi-stage detection algorithms, the YOLO-based object detection algorithms, such as YOLOv4 [21], YOLOv5 [24], and YOLOv7 [44], demonstrate significantly faster object detection speed. In recent years, some YOLO-based algorithms have been applied to flame detection. Zhao et al. [45] proposed an improved Fire-YOLO deep learning algorithm for the detection of fire targets in forest fire images. Lestari et al. [46] proposed a method that can monitor an area of fire in a building. Goyal et al. [47] used both deep learning and infrared cameras to monitor the forest and surrounding area. Xiao et al. utilized the YOLOv5 deep neural network to develop a detection system for early fire warning in monitoring substations [48]. They are very effective in detecting static flames. However, when confronted with dynamic flames, this type of YOLO-based algorithms perform poorly in terms of detection precision since the landscape of flames varies over time.

Recently, a growing number of scholars have shifted their focus to dynamic flames. For instance, Avazov et al. [49] proposed a method that relies on a lightweight CNN model and an enhanced version of YOLOv3 for detecting dynamic flames in shipyard areas. Li et al. [50] concentrated on utilizing diverse motion detection methods, such as adaptive background subtraction and motion history images, for the effective identification of dynamic flames. Wang et al. [51] employed a diverse set of methodologies, incorporating visualized heat release rate prediction, root mean square error and mean absolute error comparisons, as well as an analysis of detection time, to enhance the accuracy and efficiency of dynamic flame detection systems. Despite the effectiveness of these methods in detecting dynamic flames, they often overlook the crucial aspect of precisely locating the root region of the flame, which is a more effective area for firefighting purposes.

Based on existing literature, current research in flame recognition has primarily focused on balancing model accuracy and efficiency, often overlooking the enhancement of robustness in flame detection models. However, recent studies [52–54] suggest that the robustness of object detection models is a critical factor in designing new models, and the temporal consistency-based detection (TCD) algorithms perform excellent in improving robustness. This type of algorithm ensures that the recognized flames do not change abruptly in successive frames. TCD algorithms are commonly used in computer vision [30, 55]. These algorithms aim to enhance the precision and robustness of detecting objects by leveraging the temporal information presented in consecutive frames of a video sequence. Nishimura et al. [52] proposed a semi-supervised cell-detection method that uses a time-lapse sequence. Jeong et al. [54] introduced a consistency-based semi-supervised learning approach for object detection. Xiao et al. [53] presented a method for detecting adversarial frames based on the temporal consistency property of videos. They focus on ensuring that detected objects demonstrate a consistent appearance and behavior of motion across multiple frames. Compared to other object detection algorithms, these methods exhibit an improved capability to differentiate between truly objects and false detections by taking into account the temporal context.

To further demonstrate the characteristics of the related studies, a summary of the algorithms related to the proposed DDAF is given in Table 1. It can be seen that the research on flame detection has not been designed based on the two types of key features of flame, i.e., dynamic recognition and flame root region localization. More importantly, the existing flame detection methods have not explicitly considered how to improve the robustness, however, the research on robustness receives widespread attention on other object detection scenarios. Therefore, this paper takes these three limitations as the starting points to designed an effective object detection framework tailored for dynamic flame root region detection. Specifically, the integration of DCNv2, CAM, and DH effectively extracts flame features. Moreover, the pruning technique significantly improves flame detection speed. IM achieves notable success in flame root region localization and TCD enhances flame detection robustness.

**Table 1:** Summary of the related works.

Author	Year	Algorithm Type	One-Stage	Flame Type	Central Location	Core Work
Settersten et al. [40]	2002	Conventional	No	Static	None	Using two-color polarization spectroscopy and two-color resonant four-wave mixing techniques to detect flame.
Hao [4]	2007	Conventional	No	Static	None	Utilizing a laser generator or projector to project straight lines on the measuring.
Liu et al. [6]	2011	Conventional	No	Static	None	Using VID detectors to identify fires, reflections, hot surfaces, and smoke characteristics;
Cheong et al. [38]	2011	Conventional	No	Static	None	Using sensor network node for UV flame detection.
Genovese et al. [8]	2011	Conventional	No	Static	None	Utilizing an infrared-ultraviolet camera to detect flames.
Weiler et al. [5]	2012	Conventional	No	Static	None	The technology of flame detection involves a combination of IR/IR methods.
Taylor et al. [37]	2013	Conventional	No	Static	None	Using signal amplification in the mid-IR.
						Utilizing multichannel IR studies for obtaining information on fire size and temperature, and developing techniques for quantitative fire evaluation.
Avendano et al. [39]	2021	Conventional	No	Static	None	Using a solar blind deep ultraviolet active pixel sensor.
Truong et al. [7]	2023	Conventional	No	Static	None	Utilizing collect the ultraviolet radiation emitted by the flame into a detector.
Lestari et al. [46]	2019	YOLO-based	Yes	Static	None	Detection fire in CCTV videos with YOLO-based.
Goyal et al. [47]	2020	YOLO-based	Yes	Static	None	Using both deep learning and infrared cameras to monitor the forest.
Li et al. [50]	2021	YOLO-based	Yes	Dynamic	None	Using GMM-based background subtraction and motion history images;
						Using color models in YCbCr and RGB spaces.
Xiao et al. [48]	2022	YOLO-based	Yes	Static	None	Using multi-feature integration technology, adaptive mixture Gaussian model, and motion memory matrix.
Zhao et al. [45]	2022	YOLO-based	Yes	Static	None	Extending feature extraction networks in three dimensions for small target object detection.
Wang et al. [51]	2022	YOLO-based	Yes	Dynamic	None	Using vHRR prediction, comparison of RMSE and MAE, and detection time analyzing to detect dynamic flames;
Avazov et al. [49]	2023	YOLO-based	Yes	Dynamic	None	Improved YOLO-v3 video image flame.
Jeong et al. [54]	2019	TCD -based	Yes	None	None	Utilizing consistency constraints as a tool;
						Improving detection performance with available unlabeled data.
Xiao et al. [53]	2019	TCD -based	Yes	None	None	An adversarial frame identifier called AdvIT based on temporal consistency in videos.
Nishimura et al. [52]	2021	TCD -based	Yes	None	None	Semi-supervised cellular assays using time series.
**This study**	2024	YOLO-based	Yes	Dynamic	Yes	Incorporating DCNv2, CAM, pruning technique, and inductive modeling.

Note: Conventional: conventional algorithm in OFDs; Static: static flame; Dynamic: dynamic flame.

### 2.2 Background techniques

#### 2.2.1 Upsample

**Fig 1:**
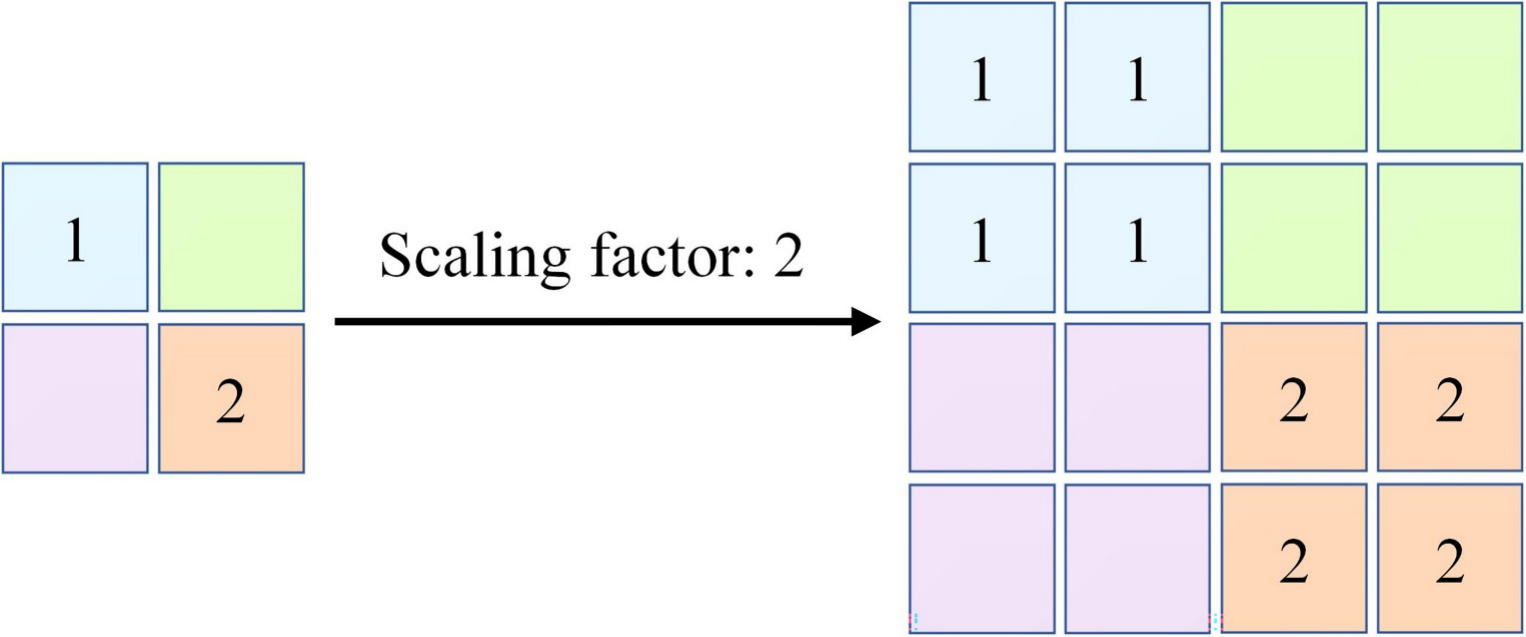
Example diagram of the upsample process.

Upsample is a technique that used to map low-resolution images to higher resolutions. This study employs nearest-neighbor interpolation for the up-sampling process (details are shown in Fig 1). Given an original image *F* with dimensions *M* × *N* and a desired up-sampled size represented as *P*×*Q*(*P*>*M*,*Q*>*N*). The up-sampled image is denoted as *G* and its corresponding pixel position is denoted as *G*(*i*,*j*). This process is indispensable in DDAF, particularly in handling intricate details of images, as encountered in flame detection, expressed mathematically as follows:

G(i,j)=F(round((i−1)×(M−1)/(P−1))+1,round((j−1)×(N−1)/(Q−1))+1) (1)

where *round*(∙) denotes the process of rounding off the values, *i* and *j* represent the row and column indices of the image after upsample.

#### 2.2.2 Conv blocks

Conv blocks are consisted of residual convolutional blocks, where the convolution operation extracts feature from input data, the activation function introduces non-linear elements, and the BN layer aids in the training of the network. These blocks are defined as:

$$\begin{array}{l} \hat{X}=Siou(BN(Conv_{3\times 3}(X))),\\ X=BN(Conv_{3\times 3}(\hat{X}))+X, \end{array}$$ (2)

where *BN* denotes batch normalization, *Siou* represents the activation function, *Conv*_3×3_ denotes convolution operation with a kernel size of 3×3. Following the design principles, these convolutions are dense.

#### 2.2.3 Spatial pyramid pooling features

**Fig 2:**
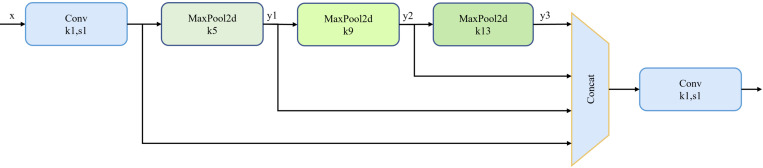
Illustrate of SPPF. Best viewed in color.

SPPF serves to perform a multi-scale pooling operation on the input feature graph to capture semantic information at different scales. Details are shown in Fig 2.

To begin with, SPPF takes the input feature map denoted as x, and applies a convolution operation to reduce the channel dimensions by half for alleviating computational load. Then MaxPool2d operations are conducted with kernel sizes of k = 5, k = 9, and k = 13 on the downsized feature maps to generate y1, y1, and y3 respectively. These feature maps capture semantic information at various scales, corresponding to different levels of detail. Subsequently, a concat operation is executed to concatenate the original feature map x with y1, y2, and y3. Finally, a k = 1×1 convolution operation is performed to adjust the channel dimensions of the concatenated feature maps.

## 3 Dynamic Deformable Adaptive Framework (DDAF)

Xu et al. (2019) proposed a method that includes adaptive spatial feature selection and temporal consistency constraints, enabling joint spatial-temporal filter learning in a lower-dimensional discriminative manifold. Inspired by this work, this paper proposes the DDAF framework. It aims to mitigate the limitations of slow and false detection of conventional OFDs, as well as the detection of dynamic flames varies over time.

### 3.1 Architecture

**Fig 3:**
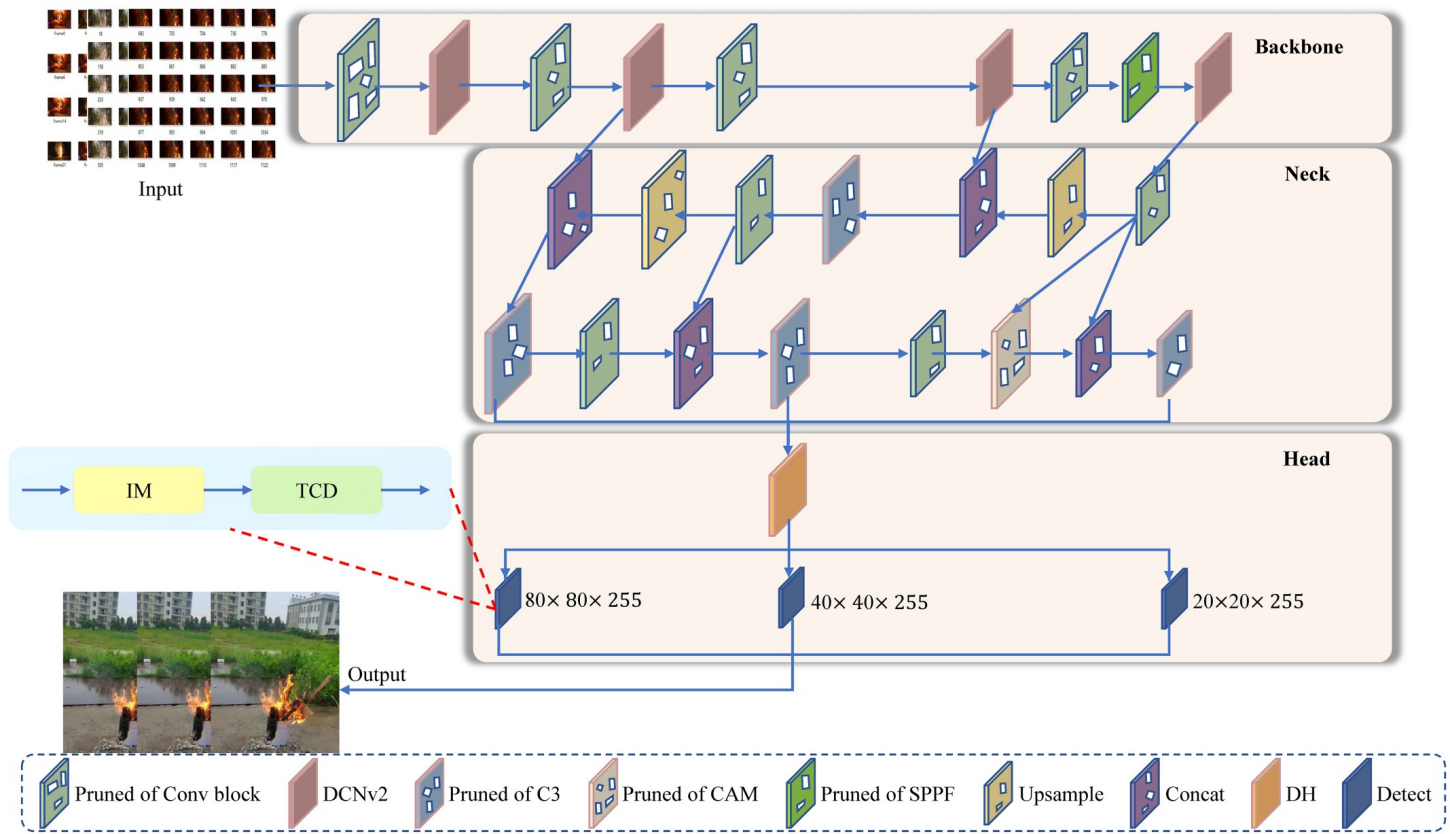
Illustrate of the DDAF. This study primarily uses DCNv2 for feature extraction, CAM for fusion, and DH for dynamic flame detection. Additional details are presented in the backbone, neck, and head. Best viewed in color.

As shown in Fig 3, the image of size 640*640 is fed at the input. The DDAF framework consists of three parts: Backbone, Neck, and Head. They are responsible for extracting flame features, enhancing and fusing these features, and generating target detection results, respectively. In the Backbone network, the DCNv2 structure enables the network to extract features at different scales, followed by the concatenate (Concat) operation, and finally processes the output feature vector using SPPF. The Neck network uses FPN+PAN+CAM structure for the adaptive fusion of deep and shallow network features, thus improving the quality of features extracted from the target. The feature pyramid network (FPN) [56] structure passes deep semantic features downward from top to bottom (top-down). It effectively utilizes multi-scale feature maps for improved precision and robustness in detecting objects of different sizes within an image. The pixel aggregation network (PAN) [57] structure bottom to up (bottom-up) complements the FPN by passing the low-level localization features upward. Then, the CAM integration into FPN performs an adaptive fusion of features by using expanded convolution with varying rates. The integration of FPN, PAN, and CAM structures into the Neck network enables adaptive fusion, leveraging multi-scale features, complementing low-level features, and adaptively merging features to improve the accuracy and robustness of flame detection. The Head network employs dynamic head (DH) detection to improve the detection of dynamic flames effectively, then combines IM and temporal consistency-based detection to achieve stable root region localization. Eventually, a pruning algorithm is used to improve the detection speed of the model. In summary, the deformable convolutional network v2 (DCNv2) contributes to flexible feature extraction, while the Context Augmentation Module (CAM) enhances contextual information. The Dynamic Head (DH) provides effective detection of dynamic flames, complemented by Inductive Modeling (IM) and Temporal Consistency-based Detection (TCD) for stable root region localization. Together, these components ensure a comprehensive and adaptive approach to dynamic real-time flame detection.

### 3.2 Deformable convolution network v2

**Fig 4:**
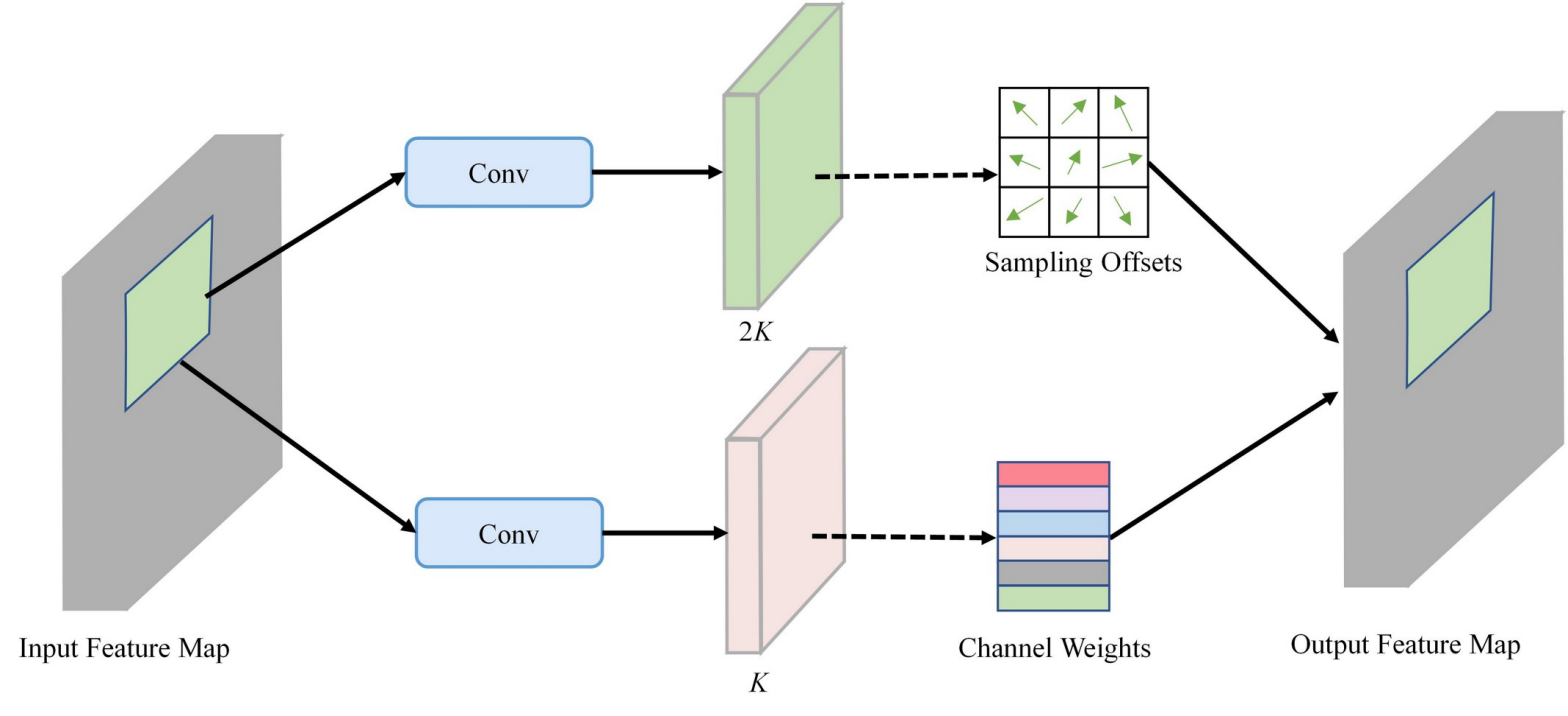
Illustrate of deformable convolutional. Input feature map with 2*K* and *K* output channels. The 2*K* portion represents sampling offsets, and the *K* portion represents channel weights. The final aggregation results in the output feature map. Best viewed in color.

DCNv2 [27] introduces the modulation mechanism into the standard deformable module [58]. This modulation mechanism allows convolutional kernels to dynamically adjust their shape based on input features, thereby improving flexibility in capturing spatial details and enhancing feature extraction capabilities, and the modulated deformable convolution is reformulated as:

$$y(p)=\sum_{k=1}^{K}w_{k}\cdot x(p+p_{k}+\Delta p_{k})\cdot \Delta m_{k}$$ (3)

where Δ*p*_*k*_ and Δ*m*_*k*_ are the learnable offset and modulation scalar for the *k*-th location, respectively. As shown in Fig 4, both the offset Δ*p*_*k*_ and modulation Δ*m*_*k*_ are obtained via a separate convolutional layer applied over the same input feature maps *x* with 2*K* and *K* output channels respectively. Additionally, to enhance the model of ability for geometric transformation, DCNv2 replaces 10 more plain counterparts than the setting of DCNv1 in the ResNet [59] with deformable convolution.

### 3.3 Context augmentation module

**Fig 5:**
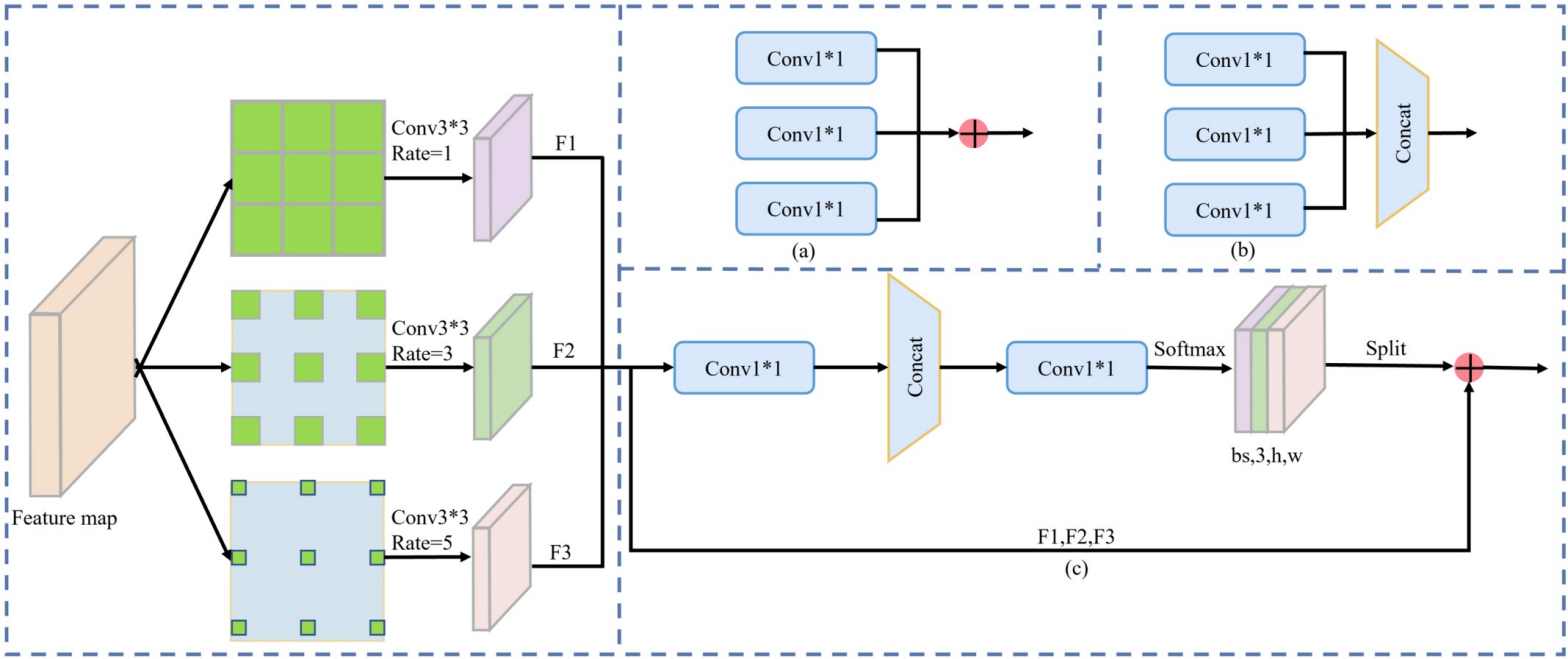
Illustrate the adaptive fusion model. Best viewed in color. Method (a) and (b) are weighted fusion and concatenation operation respectively. Method (c) is an adaptive fusion method.

The small landscape of the flame is difficult to detect as usual, necessitating a wealth of feature information. In this paper, in order to enhance the information fusion of different feature layers for tiny objects, the CAM [28] structure is added. The CAM structure is improved from the FPN structure, and the main function is to adaptively (c) learn the weights of feature fusion across various levels. The details of CAM can be found in Fig 5.

The incorporation of the CAM (Contextual Attention Module) structure is an enhancement for information fusion. By employing dilated convolutions with rates of 1, 3, and 5, the CAM effectively broadens its receptive field. The rationale behind using dilated convolutions lies in their ability to increase the convolutional kernel’s receptive field, thereby enhancing the model’s capacity to capture contextual information. This deliberate expansion of the receptive field is essential for overcoming the challenge of detecting small flames, as it enables the model to gather spatial context and intricate details associated with varying flame sizes. The kernel size is 3×3, and the rates are 1, 3, and 5. The (c) is an adaptive fusion method. Specifically, assuming the input has a size of (bs, C, H, W), convolution operations can produce spatial adaptive weights with a shape of (bs, 3, H, W). Methods (a) and (b) are weighted fusion and concatenation of channel dimensions, respectively. The CAM effectively addresses the challenge of detecting small flames by capturing spatial context through varying receptive fields.

### 3.4 Dynamic head

**Fig 6:**
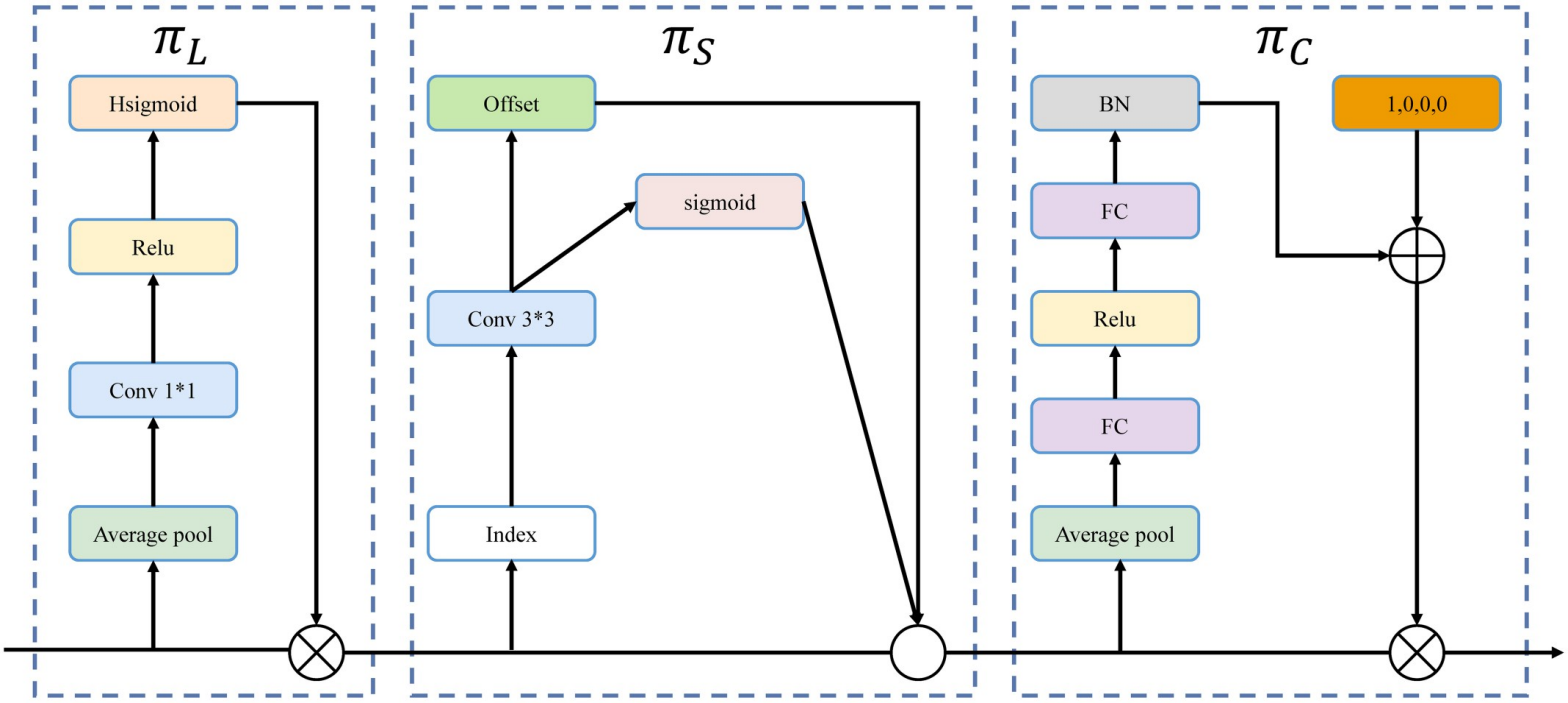
Structure of DH block.

The FPN is a detection structure that combines multi-scale convolution features. However, during the down-sampling process, there is potential for information loss in detecting small landscape flame targets. In contrast, a method known as DH [29] can effectively mitigate this limitation. Specifically, the method redefines the four-dimensional tensor *L*×*H*×*W*×*C* into a three-dimensional tensor *L*×*S*×*C*. It employs scale-aware, spatial-aware, and task-aware attention mechanisms across the *L*, *S*, and *C* dimensions to fuse features of different scales to selectively focus on key features. This key attention helps preserve essential information, especially in regions prone to loss during down-sampling. In flame detection, DH ensures precise localization of dynamic flames by effectively capturing real-time features. More detailed information can be found in Fig 6.

The feature tensor is *F* ϵ *R*^*L*×*S*×*C*^, where *L* denotes the number of pyramid layers, *S* denotes the size of the feature, and *C* denotes the number of channels. Moreover, *S* = *H*×*W*, where *H*, *W* denote the height and width of the feature. DH can be expressed as:

$$W(F)=\pi_{C}(\pi_{S}(\pi_{L}(F)\cdot F)\cdot F)\cdot F$$ (4)

where *π*_*L*_(∙), *π*_*S*_(∙), and *π*_*LC*_(∙) correspond to scale-aware attention, spatial-aware attention, and task-aware attention, respectively. *π*_*L*_ enables dynamic feature fusion based on the importance of the features in each layer. The expression is shown in Eq (3).

$$\pi_{L}(F)\cdot F=\sigma (f(\frac{1}{SC}\sum_{S,C}F))\cdot F$$ (5)

where *f*(∙) represents a 1×1 convolutional layer, and σ(x)=max(0,min(1,(x+1)/2)) denotes a H-sigmoid function.

The sparsity is first learned using deformed convolution v2 [27], then the cross-level features are aggregated at the same spatial locations. The expression is shown in Eq (4).

$$\pi_{S}(F)\cdot F=\frac{1}{L}\sum_{l=1}^{L}\sum_{k=1}^{K}\omega_{l,k}\cdot F(l;p_{k}+\Delta p_{k};c)\cdot \Delta m_{k}$$ (6)

where *K* is the number of sparse sampling positions, *p*_*k*_+Δ*p*_*k*_ is the offset position when the self-learning spatial offset Δ*p*_*k*_ is focused on a specific region, and Δ*m*_*k*_ is the significant scalar at the self-learning position *p*_*k*_. The task-aware attention module dynamically opens or closes the feature channel to select different tasks with the expression shown in Eq (5).

$$\pi_{C}(F)\cdot F=\max(\alpha^{1}(F)\cdot F_{C}+\beta^{1}(F),\alpha^{2}(F)\cdot F_{C}+\beta^{2}(F))$$ (7)

where [*α*^1^,*α*^2^,*β*^1^,*β*^2^]^*T*^ is a hyperfunction to learn control activation thresholds. It first performs a global pooling on the *L*×*S* dimensions to reduce dimensionality, then uses two fully connected layers and a normalization layer, and final normalizes by the sigmoid activation function. Global pooling facilitates the aggregation of feature maps across spatial dimensions, allowing for the capture of global context and a reduction in spatial information. The ensuing fully connected layers further transform these features into a vector, catering to classification or regression tasks. This sequence of operations enhances attention mechanisms, providing improved focus on specific perspectives, including scale, spatial relationships, and task-specific details. Consequently, this refined method contributes to an enhanced performance in flame detection. This type of attention integration module can be stacked based on Eq (5).

**Fig 7:**
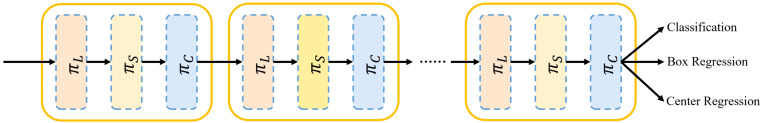
Connection scheme of DH blocks. The ***π***_***L***_, ***π***_***S***_, and ***π***_***C***_ represent scale-aware, spatial-aware, and task-aware attention, respectively.

These pyramids can be scaled to the same size 3D tensor *L*×*S*×*C*. This tensor is then fed to the dynamic detection head, which consists of several DH blocks as shown in Fig 7. The output of the DH can be used for a variety of tasks, including classification and bounding box regression. The several DH blocks are arranged in the order of *L*, *S*, and *C*. Based on the number of DH blocks, this study compares the AP_0.5_, precision, and AP_0.5:0.95_, as shown in Table 9.

### 3.5 Layer-adaptive magnitude-based pruning (LAMP)

LAMP [31] proposes a novel importance scoring method perspective for global pruning from the model-level distortion minimization. Specifically, each neural network layer can be considered as an operator for studying the model-level distortion produced by the pruned layers. Assuming the weights are sorted in ascending order according to the index map, LAMP apply it to each unexpanded vector without loss of generality, i.e., *u* ≤ *v* whenever |*W* [*u*]| ≤ |*W* [*v*]| holds, where |*W* [*u*]| denotes the *W* entry mapped by index *u*. The *u*-th LAMP score of the weight tensor *W* is defined as follows:

$$score(u;W):=\frac{{\left(W[u]\right)}^{2}}{\sum_{v\geq u}{\left(W[v]\right)}^{2}}$$ (8)

**Fig 8:**
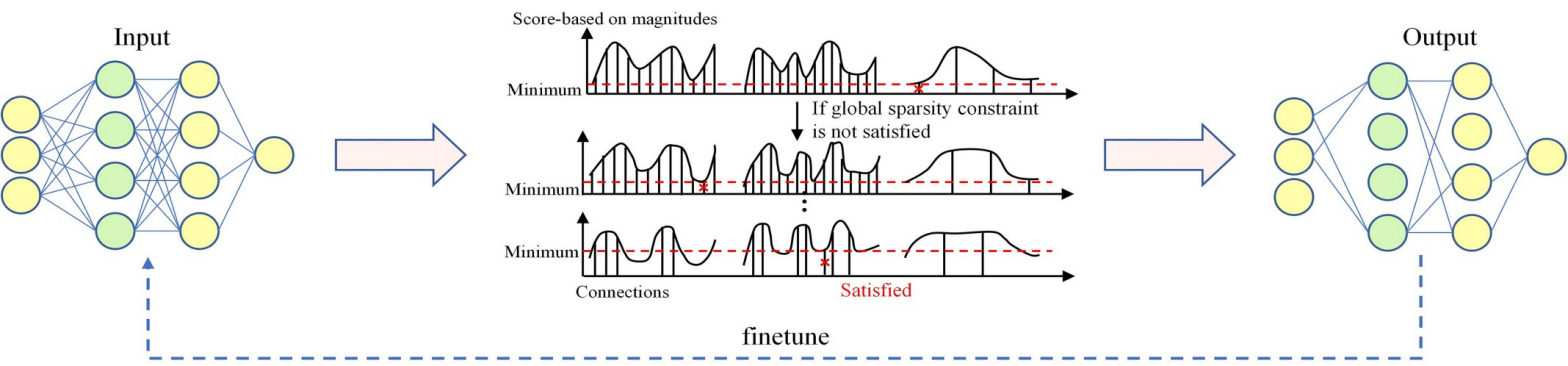
Illustration of pruning process of LAMP. First, the structured pruning is performed using the layer-adaptive magnitude-based pruning (LAMP) method where the connection with the smallest LAMP score is pruned sequentially till the required global sparsity constraint is satisfied. This method significantly reduces the quantity of parameters. Then the clipped model is trained, i.e., finetune.

Informally, the LAMP score (Eq 8) measures the relative importance of the target connection among all existing connections belonging to the same layer. Connections with smaller magnitudes (in the same layer) have been pruned. Therefore, two connections with the same weight magnitude will have different LAMP scores. Once the LAMP score is calculated, this algorithm prunes the connection with the smallest LAMP score globally until the desired global sparsity constraint is reached. The details are shown in Fig 8.

### 3.6 Inductive modeling

**Fig 9:**
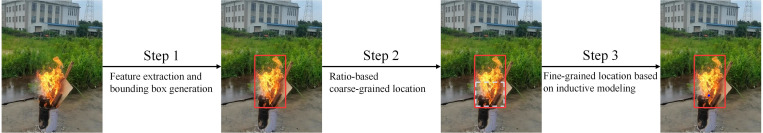
Visual representation of inductive modeling steps.

In this paper, an inductive modeling method [32] based on the position of the detection box is proposed to mitigate the flame root region localization challenge with the following steps, as shown in Fig 9:

1. Feature extraction and bounding box generation: Through the feature fusion algorithm, which includes DCNv2, CAM, and DH, the flame is detected with the aim of finding the position of the flame and the bounding box information.
2. Ratio-based coarse-grained location: Given the typical location of flames near the bottom of images, a preliminary localization of the root region within the bounding box is approximated based on this assumption.
3. Fine-grained location based on inductive modeling: Considering both the position and size of the bounding box, thus a more precise localization of the flame root region is determined in Eq (9).

$$\left\{ \begin{array}{l} m=w/\kappa +x\\ n=h/\lambda +y \end{array}\right.$$ (9)

where (*x*, *y*) are the coordinates of the upper left corner of the detection box. *w* and *h* are the width and height of the bounding box. Scaling factors 1/*κ* and 1/*λ* are applied to *w* and *h*, respectively. The flame root region is defined by coordinates (*m*, *n*), with points set at a radius of 5 pixels.

## 4 Experiments

### 4.1 Experimental setup

**Fig 10:**
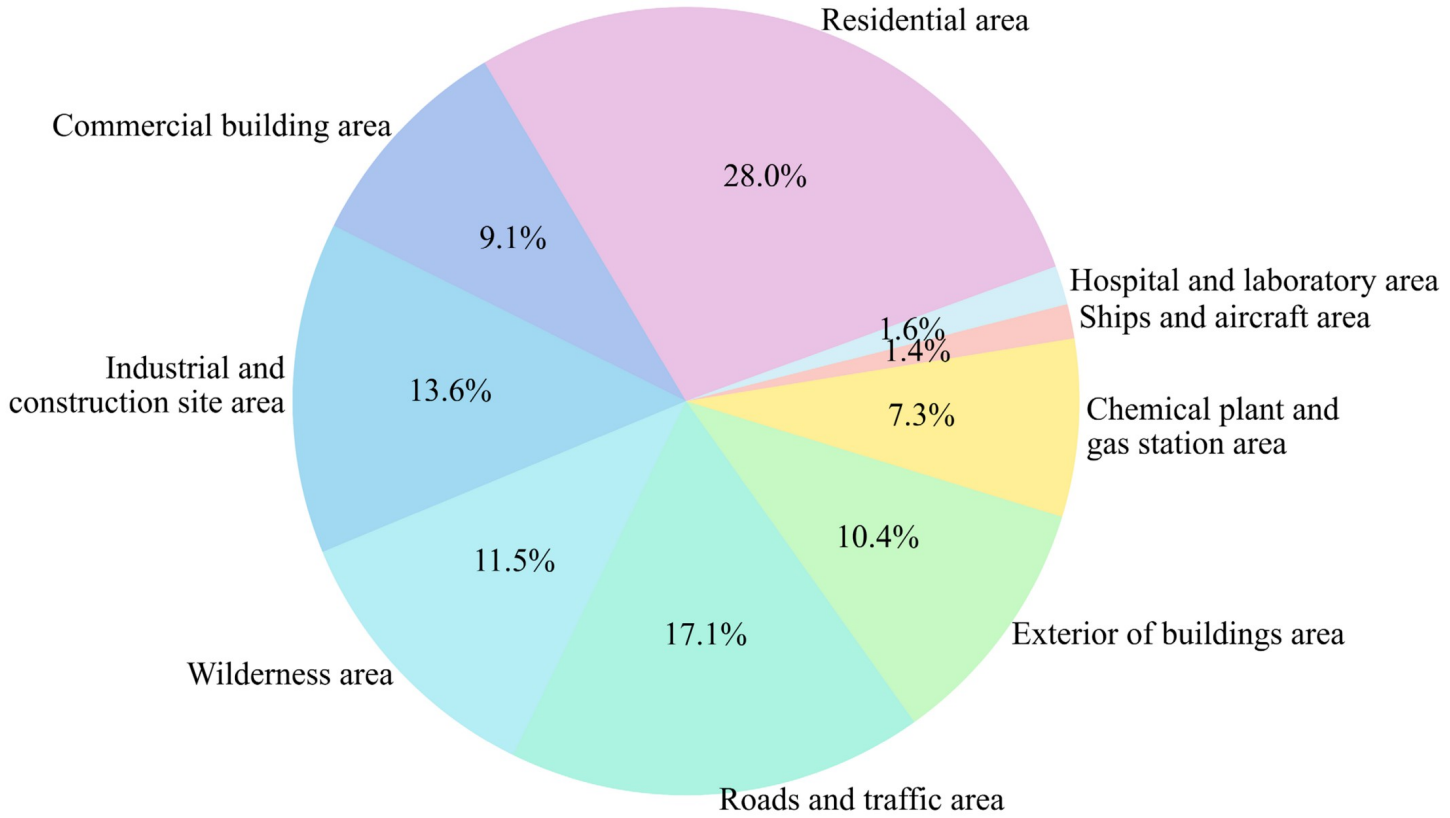
Percentage distribution of images in each category.

The dataset utilized in this study is a combination of contributions from four works, i.e. [60–63], where images of forest fires and images with flames filling the entire image are removed to validate the reliability of our flame detection method for urban fires. The flame dataset has a total of 4,248 images, with 3,811 images in the training set and 437 images in the validation set. The distribution ratio of custom dataset across different categories in shown in Fig 10. All experiments are first trained using the training set and validated using the validation set. The validation set help to determine if the model generalizes well and to assess the effectiveness of the flame detection application. All experiments are conducted under consistent environmental conditions and hyperparameters. The experimental environment is shown in Table 2.

**Table 2:** Experimental environment parameters and setting.

	Name	Parameter
Hardware	CPU	Intel i5 12400F
	GPU	NVIDIA GeForce RTX 3060 12G
Software	operating system	Windows11
	programming language	Python 3.7.16
	deep learning framework	Pytorch 1.13.1 & CUDA 11.7
Configuration	epoch	300
	batch size	16
	initial learning rate	0.01
	learning rate momentum	0.937
	weight decay	0.0005
	image size	640 × 640
	optimizer	SGD
	number works	6 training thread

### 4.2 Evaluation indexes

As shown in Eqs (10–13), the evaluation parameters which are defined in detail as follows: TP represents correctly identified positive instances, FP indicates falsely identified positives, TN denotes correctly identified negatives, and FN signifies falsely identified negatives. The confusion matrix synthesizes these indexes, enabling a comprehensive evaluation of model performance by computing accuracy, recall, precision, and F1 score. recall (R) and precision (P) are calculated from the confusion matrix shown in Table 3. R is the ability of the model to successfully detect all real flames, while P indicates how many of the flames detected by the model are real flames. The weighted average summed of precision and recall can be expressed as F1 score (F1). Compared to F1, the average precision (AP) reflects the overall detection performance of the model. It can be obtained by calculating the area under the corresponding precision-recall curve. This paper also uses frames per second (FPS) to characterize the timing detection performance of the algorithm. These indexes measure the comprehensiveness and accuracy of the model in identifying flames. In general, 24 FPS must be achieved to guarantee real-time detection [64]. To minimize the effect of potential outliers or fluctuations, the frames per second (FPS) value is the average FPS value of 30 separate runs with a batch size of 1. Calculating the average FPS value of these 30 separate runs involves adding up the FPS values for each run and dividing the total value by 30.


$$P_{recision}=\frac{TP}{TP+FP}$$ (10)

$$R_{ecall}=\frac{TP}{TP+FN}$$ (11)

$$F1=\frac{2\times P_{recision}\times R_{ecall}}{P_{recision}+R_{ecall}}=\frac{2TP}{2TP\times FP\times FN}$$ (12)

$$AP=\int_{0}^{1}p(r)dr$$ (13)

**Table 3:** Confusion matrix.

Reference	Prediction	
	positive	negative
positive	TP	FN
negative	FP	TN

### 4.3 Comparison of different object detection algorithms

In order to verify the flame detection algorithm, a comparative analysis of various object detection algorithms was conducted. In this study, we selected previous versions of YOLO [21–23, 42–44] as well as the SOTA YOLOv7-tiny [44] as baselines to assess the performance of our proposed model. Moreover, the existing derived models based on these baselines are employed to further validate the effects of our proposed contributions on YOLOv5s. Table 4 presents the comparison results between the proposed DDAF model and these compared models trained under the same settings.

**Table 4:** The results of different object detection algorithms.

Model	AP_0.5_	AP_0.5:0.95_	P	R	F1	FPS
yolov3-tiny	0.734 (+ 10.9%)	0.411 (+ 20.0%)	0.764 (+ 5.5%)	0.743 (+ 1.6%)	0.753 (+ 3.6%)	**198.219** (- 87.5%)
yolov4-tiny	0.765 (+ 6.4%)	0.413 (+ 19.4%)	0.775 (+ 4.0%)	0.739 (+ 2.2%)	0.757 (+ 3.0%)	180.415 (- 86.2%)
yolov4-tiny-3	0.626 (+ 30.0%)	0.296 (+ 66.6%)	0.639 (+ 26.1%)	0.608 (+ 24.2%)	0.623 (+ 25.2%)	177.558 (- 86.1%)
yolov5n	0.789 (+ 3.2%)	0.465 (+ 6.0%)	0.796 (+ 1.3%)	0.739 (+ 2.2%)	0.766 (+ 1.8%)	102.863 (- 75.9%)
yolov5n6	0.793 (+ 2.6%)	0.486 (+ 1.4%)	0.792 (+ 1.8%)	0.728 (+ 3.7%)	0.759 (+ 2.8%)	93.969 (- 73.6%)
yolov5s	0.782 (+ 4.1%)	0.478 (+ 3.1%)	0.778 (+ 3.6%)	0.756 (- 0.1%)	0.767 (+ 1.7%)	121.929 (- 79.7%)
yolov5s6	0.805 (+ 1.1%)	0.489 (+ 0.8%)	0.802 (+ 0.5%)	**0.76** (- 0.7%)	**0.78** (+ 0%)	95.715 (- 74.1%)
yolov5s-ghostl	0.8 (+ 1.8%)	0.474 (+ 4.0%)	0.789 (+ 2.2%)	**0.76** (- 0.7%)	0.774 (+ 0.8%)	70.283 (- 64.8%)
yolov5s-LeakyReLU	0.802 (+ 1.5%)	0.469 (+ 5.1%)	0.785 (+ 2.7%)	0.732 (+ 3.1%)	0.758 (+ 2.9%)	116.137 (- 78.7%)
yolov5s-transformer	0.78 (+ 4.4%)	0.456 (+ 8.1%)	0.777 (+ 3.7%)	0.74 (+ 2.0%)	0.758 (+ 2.9%)	109.766 (- 77.4%)
yolov7-tiny	0.801 (+ 1.6%)	0.477 (+ 3.4%)	**0.809** (- 0.4%)	0.736 (+ 2.6%)	0.771 (+ 1.2%)	61.35 (- 59.6%)
yolov7-tiny-Silu	0.795 (+ 2.4%)	0.483 (+ 2.1%)	0.787 (+ 2.4%)	0.757 (- 0.3%)	0.772 (+ 1.0%)	60.976 (- 59.4%)
DDAF^+^	**0.814**	**0.493**	0.806	0.755	**0.78**	24.762

Note: Assuming the proposed and compared methods are denoted as *A* and *B*, respectively, the percentage can be calculated using the formula: (*A*—*B*)/*B*.

In Table 4, comparing all the algorithms, DDAF has the best performance on AP_0.5_ and AP_0.5:0.95_ with 0.814 and 0.493, respectively. It reflects the most advantageous in flame detection precision. Moreover, on classical indexes, DDAF obtains optimal results on F1 values, only performs slightly worse to YOLOv7-tiny on P values, and is superior to most methods on R values. This shows that the DDAF performs highly competitive results on classical indexes. Although DDAF is obviously lower than other comparative algorithms in the FPS values, but it still satisfies the base speed requirements (e.g., FPS≥24) for real-time firefighting scenarios [64].

As shown in Table 6, in order to further improve the detection precision of DDAF, the data augmentation (AD) technique is integrated into DDAF* called DDAF^+^. For further enhance the model performance of DDAF, a series of pilot experiments of pruning are conducted to systematically optimize several key hyperparameters such as speed up, finetune epochs, and learning rates. Other detailed experimental configurations are provided in Table 5. Table 6 shows the results of the comparison for DDAF*, DDAF^+^ and DDAF.

**Table 5:** Pruning parameter configuration.

Params	Batch-size	Epochs		Speed up
		Normal training	Finetune	
Value	16	300	230	1.5

**Table 6:** The results of different object detection algorithms.

	Params(M)	FLOPs (G)	AP_0.5_	AP_0.5:0.95_	P	R	F1	FPS
DDAF*	10.28 (- 49.0%)	17.6 (- 33.5%)	0.814 (+ 1.5%)	0.493 (+ 0.4%)	0.806 (- 0.2%)	0.755 (+ 1.2%)	0.78 (+ 0.4%)	24.762 (+ 261.8%)
DDAF^+^	10.28 (- 49.0%)	17.6 (- 33.5%)	0.82 (+ 0.7%)	**0.5** (- 1.0%)	**0.808** (- 0.5%)	0.745 (+ 2.6%)	0.775 (+ 1.0%)	24.142 (+ 271.1%)
DDAF	5.24	11.7	**0.826**	0.495	0.804	**0.764**	**0.783**	**89.6**

Note: Assuming the proposed and compared methods are denoted as *A* and *B*, respectively, the percentage can be calculated using the formula: (*A*—*B*)/*B*.

In Table 6, the detection precision of DDAF^+^ is improved to 0.82. However, the limitation on the FPS value has not alleviated. Expanding on this groundwork, we further incorporated pruning technique, resulting in a detection speed of 89.6 FPS, with substantial enhancements in both parameter and FLOPs reduced. Compared to DDAF*, the FPS of DDAF is improved by 261.8%. It’s worth noting that this advanced algorithm is referred to as DDAF. The results show that the AP_0.5_ on our custom dataset is increased to 0.826 while concurrently reducing parameters and FLOPs by 49.0% and 33.5%, respectively. In summary, the DDAF algorithm has achieved a great balance between detection precision and speed.

### 4.4 Ablation study

In order to verify the effectiveness of each method proposed in this paper, ablation experiments were conducted and the results are shown in Table 7. DCNv2, CAM and DH are the three main components of DDAF. We add them to the baseline incrementally to compare the effectiveness of each component.

**Table 7:** Ablation comparison of model performance improvement on the custom dataset.

YOLOv5s	DCNv2	CAM	DH	AP_0.5_	AP_0.5:0.95_	P	R	F1	FPS
**✓**				0.782	0.478	0.778	0.756	0.767	121.929
**✓**	**✓**			0.795	0.48	0.791	0.763	0.777	115.342
**✓**		**✓**		0.796	0.475	0.803	0.75	0.776	107.579
**✓**			**✓**	0.813	0.49	0.786	**0.775**	0.78	25.693
**✓**	**✓**	**✓**		0.812	0.484	0.797	0.752	0.774	91.033
**✓**	**✓**		**✓**	0.804	0.486	0.802	0.743	0.771	54.586
**✓**		**✓**	**✓**	0.808	0.485	0.804	0.764	**0.783**	60.452
**✓**	**✓**	**✓**	**✓**	**0.814**	**0.493**	**0.806**	0.755	0.78	24.762

Different components have similar effects. For example, both DCNv2 and CAM are scale-aware. Compared to DCNv2 and CAM, their combination improved 2.14% AP_0.5_ and 2.01% AP_0.5:0.95_, respectively. DCNv2 and DH are both sensitive to different objects, especially medium and large objects, and the combination of the two reached the second highest 0.804 AP_0.5_, which is a little bit lower than that of DH. The combination of CAM and DH achieved not only a 0.808 AP_0.5_ but also the second-highest precision. Each of these three components has its own strengths and weaknesses. It is evident that the overall performance, particularly in terms of AP, is optimized when all three components are utilized together. The experiment proves that compared with the baseline, DDAF comprehensively improves the average accuracy of targets at different scales, and can effectively improve the detection of small-scale targets. In conclusion, DDAF can effectively improve the real-time detection precision of dynamic flames.

Compared to the baseline, DCNv2 improves AP_0.5_ and AP_0.5:0.95_ by 1.66% and 0.42%, respectively. The reason is that DCNv2 allows each pixel in the input feature map to have an adaptive receptive field. This adaptation allows for better capture of complex object details and handling variations in object shape and size. However, above the FPS, it is about 14 frames lower than the baseline. On the one hand, the increased model parameters result in the need for more computation and storage requirements. On the other hand, increased memory and bandwidth requirements lead to slower data transfer.

The CAM also achieves good performance with its performance being 1.79% AP_0.5_ and, 3.21% P and FPS slightly lower than the baseline while its FLOPs are minimal. The mechanism of adaptive expanded convolution fusion reduces the computational burden of the model. However, the performance of CAM on AP_0.5:0.95_ performs only about the same as the baseline. The reason for this is that performing expanded convolution to select different rates discards some of the valid information, thus reducing part of the model’s ability to capture details in the image.

The DH we utilized had the most significant benefits of all the components. Increasing DH compared to the baseline improves AP_0.5_ and AP_0.5:0.95_ by 3.96% and 2.51%, respectively. This is due to the fact that the three attention mechanisms adaptively fuse multiple layers of features, which enhances the ability of the probe head to discriminate different feature points, thus improving the perceptual ability of the model. At the same time, DH also brings about a serious reduction in FPS, which is due to the inclusion of additional learnable parameters, i.e., newly added weights and biases, which require more memory to store it.

#### 4.4.1 Ablation experiments for CAM fusion models

In order to verify that the adaptive module is more applicable on Head, this study compares it with the two modules of weighting and splicing on Backbone and Head respectively. The experimental results are shown in Table 8. Although the performance of the adaptive fusion model is best on Backbone, the parameter and FLOPs are too large, which results in decreasing the detection speed of the algorithm. The performance of the adaptive fusion model on Head is only slightly lower than that on Backbone. Therefore, we choose to use the adaptive fusion model on Head.

**Table 8:** Compare these three fusion models on Head and Backbone.

Add location	Models	Params(M)	FLOPs (G)	AP_0.5_	AP_0.5:0.95_	P
Baseline (YOLOv5s)		7.01	15.8	0.782	0.478	0.778
Head	weight	2.25	4.5	0.792	0.471	0.801
	concat	2.26	4.5	0.788	0.469	0.799
	adaptive	2.25	4.5	0.796	0.475	0.803
Backbone	weight	14.23	21.5	0.793	0.468	0.802
	concat	14.25	21.5	0.795	0.476	0.807
	adaptive	14.23	21.5	**0.797**	**0.482**	**0.808**

#### 4.4.2 Ablation experiments for the number of DH block stacks

This section explores the best detection results by controlling the number of stacked DH blocks. On the home-made dataset, we found that using 8 DH blocks produced the best results. We designed the following experiments using 2, 4, 6 and 8 DH blocks for comparison. As shown in Table 9, it is clear that the highest performance improvement is obtained by using 8 DH blocks. Therefore, DDAF chose 8 DH blocks.

**Table 9:** Compare the way these four modules are added.

Number blocks	AP_0.5_	AP_0.5:0.95_
Baseline (YOLOv5s)	0.782	0.478
2	0.792	0.468
4	0.808	0.485
6	0.803	0.488
8	**0.811**	**0.490**

#### 4.4.3 Ablation experiments for data augmentation types

As shown in Table 10, we found during our experiments that the model continues to improve in terms of our model performance with the moderate data augmentation approach in YOLOv5s. The hyperparameters for all three data enhancements are YOLOv5s defaults.

**Table 10:** Compare these three models.

	AP_0.5_	AP_0.5:0.95_	Precision
Baseline (DDAF*)	0.814	0.493	0.806
Scratch-med	**0.82**	**0.5**	**0.808**
Scratch-high	0.775	0.445	0.749

### 4.5 Experiment for temporally consistent video processing

**Fig 11:**
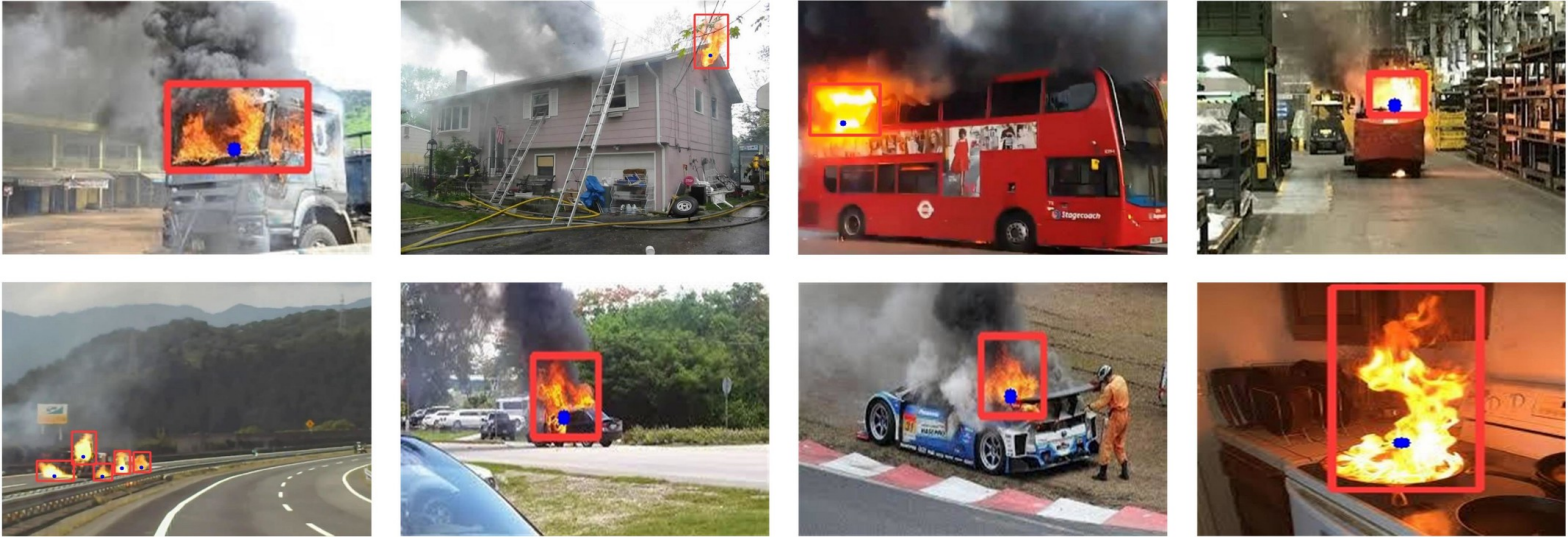
Output.

The aim of this section is to determine whether a target is real or not by focusing on the consistency of the target’s appearance and motion of the detected object in consecutive video frames. Specifically, if the same target is consistently detected for a certain number of identical images (consecutive frames) in this consecutive time frame (jumping frames) it is recognized as the flame to be detected, and conversely, if it is not consistently detected for a certain number of frames inside this number of frames it goes into a loop for the next number of frames. We performed try and error on the flame video with the parameter settings of the number of consecutive time frames and the number of continuously detected frames, and the experiments showed that the best flame detection performance in the outdoor scene was achieved when the number of jumping frames and the number of consecutive frames were set to 30 frames and 10 frames, respectively. The experimental results are shown in Fig 11.

### 4.6 Try and error for IM

In order to systematically explore the flame root region, we integrated the previous flame literature based on inductive modelling to derive a range of regions for *κ* and *λ*. We also used a stepwise approach to iteratively experiment with a range of values for *κ* and *λ*. Specifically, the values of *κ* were taken in the range of [1.8, 2.2] in increments of 0.1, and the values of *λ* were taken in the range of [1, 2], again in increments of 0.1. This exhaustive search strategy provided a comprehensive assessment of root region localization through a thorough examination of multiple parameter combinations. We conducted experiments on 437 images of the validation set and the experimental results yielded that *κ* = 2, *λ* = 1.3 is the most reasonable. This method was able to precisely mark the flame root area during the detection stage, and the picture below shows the test we did in a real scenario. The experimental results are shown in Fig 11.

## 5 Conclusions

This paper proposed an algorithm called DDAF to mitigate the limitations of the algorithms in traditional optical flame detectors (OFDs) where dynamic real-time flame detection was achieved by integrating four techniques, i.e., deformable convolution network v2 (DCNv2), context augmentation module (CAM), dynamic head (DH), and temporal consistency-based detection (TCD) into yolov5s. The pruning method of LAMP was also used to lighten the model and improve the model detection speed.

This study compared the performance of different object detection algorithms. The results showed that under the same setup conditions, the detection accuracy of DDAF was better than all the compared algorithms, and the number of parameters and FLOPs were reduced by 49.0% and 33.5%, respectively. The method used in this paper achieved a good balance between detection performance and detection speed. The proposed technology framework used in this paper was also applicable to other tasks that had special requirements for flame detection, such as wildfire monitoring, industrial safety, and combustion process control.

In the future, the construction of standard flame datasets for different firefighting scenarios may be more suitable for validating the generalization ability of the algorithm. The hyperparameters can be further optimized to improve the accuracy of flame detection. Some special noise images should be added to the training set to further validate the robustness of the algorithm. Moreover, the iterative improvement techniques may be more suitable to merged into DDAF for providing efficient adaption to changing technological environment or emerging challenges. Furthermore, other advanced pruning or knowledge distillation techniques may also able to provide efficient abilities in lightweight.
